# Photoreceptor proliferation and dysregulation of cell cycle genes in early onset inherited retinal degenerations

**DOI:** 10.1186/s12864-016-2477-9

**Published:** 2016-03-11

**Authors:** Kristin L. Gardiner, Louise Downs, Agnes I. Berta-Antalics, Evelyn Santana, Gustavo D. Aguirre, Sem Genini

**Affiliations:** Section of Ophthalmology, Department of Clinical Studies, School of Veterinary Medicine, University of Pennsylvania, 3900 Delancey Street, Philadelphia, PA 19104 USA; Augenklinik Uniklinik Erlangen, Schwabachanlage 6, 91054 Erlangen, Germany

**Keywords:** Canine models, Cell cycle, erd, Hippo pathway, Photoreceptor degeneration, Photoreceptor mitosis, rcd1, xlpra2

## Abstract

**Background:**

Mitotic terminally differentiated photoreceptors (PRs) are observed in early retinal degeneration (erd), an inherited canine retinal disease driven by mutations in the NDR kinase *STK38L* (*NDR2*).

**Results:**

We demonstrate that a similar proliferative response, but of lower magnitude, occurs in two other early onset disease models, X-linked progressive retinal atrophy 2 (xlpra2) and rod cone dysplasia 1 (rcd1). Proliferating cells are rod PRs, and not microglia or Müller cells. Expression of the cell cycle related genes *RB1* and *E2F1* as well as *CDK2,4,6* was up-regulated, but changes were mutation-specific. Changes in cyclin expression differed across all genes, diseases and time points analyzed, although *CCNA1* and *CCNE1* expression increased with age in the three models suggesting that there is a dysregulation of cell cycle gene expression in all three diseases. Unique to erd, however, are mutation-specific changes in the expression of NDR kinases and Hippo signaling members with increased expression of *MOB1* and *LATS1* in the newly generated hybrid rod/S-cones.

**Conclusions:**

Our data raise the intriguing possibility that terminally differentiated normal PRs are kept from dividing by NDR2-MOB1 interaction. Furthermore, they provide the framework for the selection of candidate genes for further investigation as potential targets of therapy.

**Electronic supplementary material:**

The online version of this article (doi:10.1186/s12864-016-2477-9) contains supplementary material, which is available to authorized users.

## Background

Photoreceptor (PR) cells are specialized retinal neurons that efficiently capture light and transduce it into a neural signal. Their intricate and highly specific structure is dependent on the expression of multiple genes, including those involved in PR specification, differentiation and maintenance [1]. Indeed, ~300 genes and loci are involved in retinal degeneration in man (RetNet: http://www.sph.uth.tmc.edu/RetNet/; October 2015), and a lower but still substantial number of genes in animals [2, 3]. While mutations in PR-specific or enriched genes are common, and cause a broad spectrum of inherited retinal diseases (reviewed by [4–6]), an understanding of the mechanistic links between mutation and disease is limited. Moreover, while apoptotic cell death is the final common pathway in most retinal degenerative diseases [7], the rate of degeneration and cell death pathway utilized varies in a disease, mutation and species-specific manner, demonstrating a high level of complexity [8–13]. Identifying the principal molecular players and providing an in-depth knowledge of the mechanisms that induce and regulate the underlying PR cell death will provide essential insight into disease progression. This will help to develop pharmacological agents and specific therapies that provide PR protection in the hopes of treating inherited retinopathies.

While stimulating the proliferation of diseased yet functional PRs to maintain the PR layer would likely be therapeutically beneficial in some retinal degenerative diseases, the proliferative potential of PR cells is controversial, and not well understood. PRs are terminally differentiated, and undergo their final cell division just prior to cell fate specification; compensatory neurogenesis mechanisms do not exist to replace all dying cells in the normal retina [1]. Indeed, it was originally postulated that new PRs are generated in naturally occurring retinal diseases, however this hypothesis has been disproved [14]. Furthermore, a recent comprehensive analysis of different rodent retinal mutants, along with a light-induced retinal degeneration model, clearly demonstrated that reactivation of the expression of cell cycle genes did not correlate with PR cell division as determined by ethynyl deoxyuridine incorporation and phospho histone H3 (PHH3) labeling. Instead, this process was essential to promote the cell death pathways [15]. In contrast, under some specific circumstances [16], a limited number of PRs and other neurons can be generated from presumably terminally differentiated Müller cells that dedifferentiate, proliferate and express neuronal progenitor markers in the adult rat, mouse as well as chicken and fish (see for review [17]).

In contrast to normal PRs and other disease models, we recently reported that a mutation in the NDR family kinase *STK38L* (*NDR2*) found in the erd canine model results in a period of sustained PR proliferation, and that newly generated cells are unique hybrid rod/S-cones [18], and did not originate from resident retinal stem cells. The *STK38L* mutation eliminates the binding sites for regulatory proteins S100B and MOB, and part of the N-terminal regulatory region that is highly conserved in all NDR subclass of AGC protein kinases [19]. NDR kinases, including LATS1, interact with the Hippo pathway through MOB1 binding to regulate aspects of cell growth, metabolism, proliferation and survival [20, 21]. Thus, we hypothesize that terminally differentiated normal PRs are kept from dividing by NDR2-MOB1 interaction, and removing this control in mutants allows the cell to re-enter the cell cycle and divide [18].

In the present study, we examined whether PR proliferation may also occur in other early-onset inherited retinal diseases to determine if common molecular pathways were involved. In addition to erd, where no equivalent disease has been reported in man [22], two other early onset canine diseases with comparable cell death kinetics and histopathology were examined: X-linked progressive retinal atrophy 2 (xlpra2) and rod cone dysplasia 1 (rcd1), which are caused, respectively, by mutations in *RPGR*ORF15 [23] and *PDE6B* [24]. Both diseases bear mutations in genes that cause human inherited blindness, and the disease phenotypes are similar and comparable. In all three diseases, the early and rapid degeneration of the PRs makes the disease course predictable and highly suitable for comparative studies of the involved events. However, the exact mechanisms by which mutations in these genes drive the degeneration events are currently unknown.

To this end, we examined the retinal and retinal pigment epithelium (RPE) expression of selected genes and proteins that are involved in cell cycle regulation, or belong to the NDR protein-kinase family and the Hippo pathway [15]; [21]. Notably, our results indicate that PR proliferation also occurred in xlpra2 and rcd1, but that formation of hybrid rod/S-cones is unique to erd. Furthermore, we demonstrate a concurrent dysregulation of critical cell cycle genes that were differentially expressed (DE) in all three diseases, while Hippo pathway genes were more specifically altered in erd.

## Results

### Morphology of early-onset canine retinal degeneration models

**Fig. 1:**
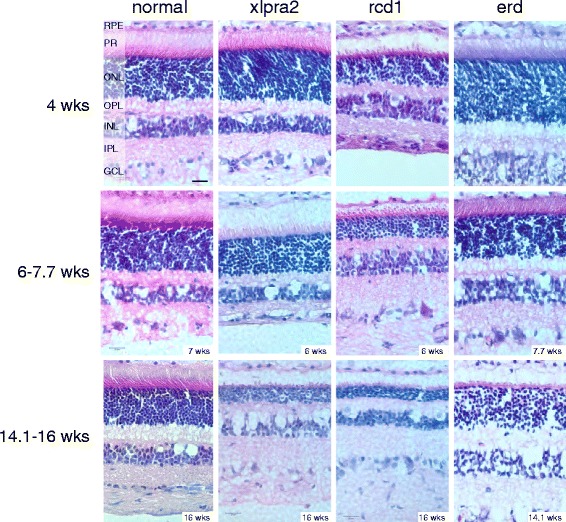
Age-dependent structural changes in normal and mutant retinas. Disease occurs earlier and progresses more rapidly in rcd1, while it is slightly delayed in xlpra2. The outer nuclear layer (ONL) in erd is preserved during the time course of the study. Scale bar: 20 μm; RPE = retinal pigment epithelium, PR = photoreceptors, ONL = outer nuclear layer, OPL = outer plexiform layer, INL = inner nuclear layer, IPL = inner plexiform layer, GCL = ganglion cell layer

We initially characterized the retinal morphology of the 3 early-onset disease models that generally have a similar pattern of PR development and degeneration (Fig. 1). Although overall retinal development is initially normal (2 wks, data not shown), there were differences in the subsequent rates and kinetics of PR degeneration; retinal degeneration started at different ages and occurred more rapidly in rcd1, where rod PR development was abnormal, and outer segments were sparse, failed to elongate, and inner segments were short already at 4 wks. The disease is slightly more delayed in xlpra2, while erd showed preservation of the ONL thickness until at least 14.1 wks.

### Photoreceptor cell proliferation in mutant retinas

**Fig. 2:**
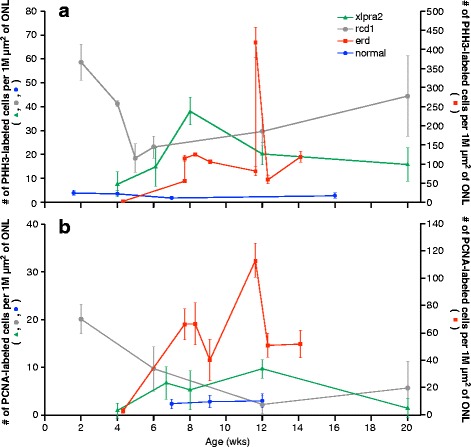
Photoreceptor cell proliferation in the outer nuclear layer of normal and mutant retinas. **a** PHH3-labeling was used to identify mitotic cells, and **b** PCNA-labeling to identify cells undergoing proliferation and DNA repair. Normal retinas exhibited essentially no mitotic cells after 2 wks of age, while they were present in the ONL of the 3 diseased retinas. Mitotic cells were continually present throughout the first 14–20 wks of life in all 3 diseases, with the highest numbers observed in erd-mutants. The PCNA data for erd has been published previously (Fig. 3c in [18]). Data points are expressed as mean of triplicates ± SD. Note the difference in the scale of the y-axes between erd (*on right*) and normal, xlpra2 and rcd1 (*left*)

To determine if PR proliferation was exclusive to erd-mutants, we used PHH3 and PCNA labeling to examine PR mitosis in the ONL of additional early-onset disease models. PHH3 is a specific marker for mitotic cells in the late G2 and M-phases [25], while PCNA labels both cells undergoing proliferation and DNA repair [26]. The number of labeled cells/1 million μm^2^ of ONL was analyzed at different time points between 2 and 20 wks. The results showed similar trends for both PHH3 and PCNA labeling in the different models and in normals (Fig. 2a and b, respectively), although the number of PCNA-positive cells was lower than the number of PHH3-positive cells at every time point examined. In addition to labeling different phases of the cell cycle, the lower PCNA results suggest that there is limited ongoing DNA damage and repair. Minimal numbers of PHH3-positive cells were found in normal retinas after 2 wks of age; these were located adjacent to the external limiting membrane and limited to the retinal periphery.

As previously shown with PCNA labeling (Fig. 2b; [18]), PHH3-labeled mitotic PRs were found at ≥7.7 wks in erd, and these levels were sustained, ranging from >50–250 cells/1 million μm^2^ of ONL. In contrast, PR proliferation peaked at 2 wks of age in rcd1 and at 8 wks in xlpra2. These time points precede (5 wks in rcd1 and 11–12 wks in erd) or approximately coincide (7 wks in xlpra2) the peaks of cell death for each disease (reviewed in [13]).

However, regardless of the disease, the number of PHH3-labeled cells in mutants far exceeded the numbers in normals, and was sustained for the time period analyzed. For example, at ~6 wks of age the mutants had 7–12 times the number of dividing cells than controls, and these increased to 12–42 times at ~8 wks.

We examined the association between cells undergoing mitosis (PHH3+), and those in mitotic checkpoint arrest (MAD2+) to exclude the possibility that entry into the late G2-M phase of the cell cycle preceded apoptosis. To this end, we compared control (6 wks) and mutant retinas at time points with high rates of PR mitosis in mutants: xlpra2 = 8 wks, rcd1 = 6 wks, erd = 8.3 wks. In the control, the PHH3 labeled ONL nuclei also labeled with MAD2; overall, the number of labeled cells with each antibody was low, and the PHH3/MAD2 ratio per 1 million μm^2^ of ONL was 1. In contrast, the PHH3/MAD2 ratio in the mutants was much higher (xlpra2 = 2.5, rcd1 = 4.2, erd = 5.9), and smaller number of nuclei/1 million μm^2^ of ONL co-expressed both proteins (xlpra2 = 9, rcd1 = 25, erd = 16). This indicates that PR mitosis is occurring in ONL, and the dividing cells contribute to maintenance of the ONL.

**Fig. 3:**
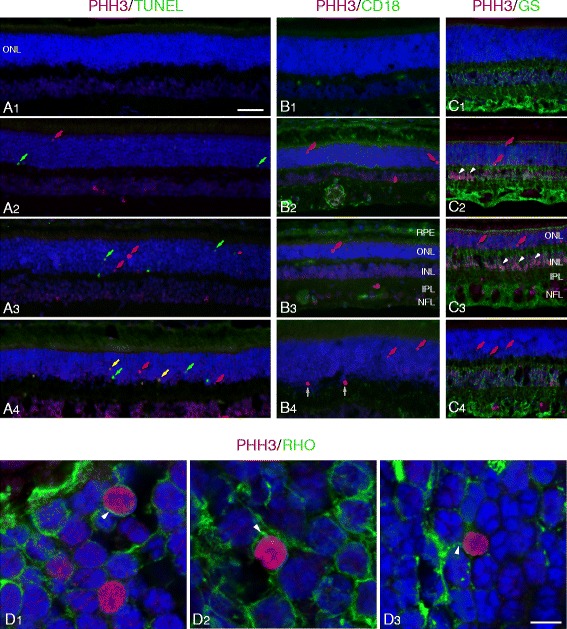
PHH3 labeling identifies mitotic cells in the outer nuclear layer. **a** Combined PHH3 (red) and TUNEL (green) labeling in normal (**A**1, 6 wks), xlpra2 (**A**2, 8 wks), rcd1 (**A**3, 6 wks) and erd (**A**4, 11.6 wks) retinas at the peak of cell death for each disease [13]. There are no TUNEL or PHH3 labeled cells in the normal retina. In the mutants, some of the red (PHH3) and green (TUNEL) labeled nuclei in ONL are identified. Note that in erd (**A**4), two cells are undergoing combined proliferation and apoptosis (yellow arrows). **b** CD18 labeling (green) shows microglial cells and processes distributed primarily in the OPL and IPL, with increased expression in xlpra2 (**B**2), rcd1 (**B**3) and erd (**B**4) compared to normal (**B**1). PHH3 labeling in the mutants shows mitotic cells in ONL (red arrows). The two dividing cells in the OPL (**B**4, white arrows) are of unknown origin and are not counted in the analysis. **c** Glutamine synthetase (GS) labeling in normal (**C**1), and dual PHH3 and GS labeling in xlpra2 (**C**2), rcd1 (**C**3) and erd (**C**4). Dividing cells in ONL (red arrows) are not labeled with GS. Note that in xlpra2 and rcd1 there are PHH3-positive nuclei in the inner border of the INL (white arrowheads). **d** Confocal microscopy image of dual-immunolabeling of retinal sections with PHH3 (red) and rod opsin (green) in the ONL of xlpra2 (**D**1), rcd1 (**D**2), and erd (**D**3). The delocalized rod opsin associated with the cell membrane forms a green halo around the labeled nuclei. RPE = retinal pigment epithelium, ONL = outer nuclear layer, INL = inner nuclear layer, IPL = inner plexiform layer, NFL = nerve fiber layer. Scale bar: A-C = 40 μm; D = 5 μm

To control for the possibility that the same PR cells were not undergoing concurrent apoptosis and mitotic division, we performed dual-labeling with TUNEL and PHH3 at the ages that correspond to the peaks of cell death for each disease [13]. The results indicate that with few exceptions the majority of cells were positive for either TUNEL or PHH3, but not for both, an indication that different cell populations in the ONL were undergoing either apoptosis or mitosis (Fig. 3A1–4). Co-localization of TUNEL and PHH3 labeling was rare and limited to few cells (1–2 per sections that extend from the optic nerve to the ora serrata) in the erd model. These results establish that proliferating and degenerating cells in the ONL are different.

### Mitotic cells in the ONL are rods

We used confocal microscopy in sections double-labeled with PHH3 and rod opsin to determine if the proliferating cells in the ONL were rods. As rod opsin delocalizes to the ONL in the early stages of disease [13], it serves as a specific marker for rods. Confocal microscopy imaging clearly demonstrated co-localization between PHH3 and rod opsin, and revealed an opsin-positive cytoplasmic halo surrounding the PHH3-positive cells in the ONL (Fig. 3D1–3). These results indicate that in the three diseases the proliferating cells in the ONL are rods. In contrast, PHH3 labeling of putative cones, i.e. the larger nuclei located immediately vitreal to the external limiting membrane, was not detected.

### Mitotic cells in the ONL are neither microglia nor Müller cells

To determine if microglia or Müller cells contributed to the pool of proliferating ONL cells observed with IHC, we undertook dual-labeling with PHH3 and markers specific for microglia (CD18; [27]) and Müller cells (glutamine synthetase (GS); [28]). CD18-labeled microglia normally reside in the inner and outer plexiform layers (IPL and OPL, respectively), and retinal diseases, in general, can result in microglial activation. However, neither microglia (Fig. 3B1–4) nor Müller cells (Fig. 3C1–4) appeared to contribute to the PHH3 positive ONL cell pool. It is important to note, however, that robust PHH3 labeling of GS positive Müller cells in the vitreal border of the inner nuclear layer (INL) was present in some areas of the rcd1 retina, and, to a lesser extent in xlpra2 (Fig. 3C2, 3); however there was no indication that these dividing Müller cells migrated to the ONL.

### Retinal gene expression

#### Age-dependent changes in normal and mutant retinas – intra-group comparisons

To properly interpret gene expression changes that occur with aging in both normals and mutants, we first performed an intra-group comparison that examined expression at time points when PR and retinal development is occurring (3 wks), when they have just reached structural maturation (5 and 7 wks) and when the retina is fully mature (16 wks) [29] (Additional file 1). Overall, the major changes in gene expression occurred between 7 vs. 3, and 16 vs. 3 wks, where many genes showed significant down-regulation compared to the previous time point within each group, suggesting that the majority of molecular events and changes take place when retinal development is still ongoing before reaching maturation (Additional file 1). With the exception of *NR2E3*, *RB1*, *E2F1*, and *CDK1*, the expression profiles were not shared between normal and mutant retinas. In the case of *CDK1*, expression was markedly up-regulated at 7 and 16 wks in normal, in rcd1 at 16 wks, and xlpra2 at 16 vs*.* 3 wks.

The expression of some cyclins decreased in normal retinas over time, as expected by the lack of cell division after two wks of age. However, this decrease was not accompanied by decreases in cyclin-dependent kinases, cell cycle phosphatases, or cyclin-dependent kinase inhibitors. In contrast, cyclin-dependent kinases, *CDK2*, *4* and *6*, decreased in expression in rcd1 at 7 compared to 3 wks; the former time point is characterized by a very high rate of apoptotic PR cell death [13]. The expression of the transcription regulators *E2F1* and *RB* was lower at all time points examined in xlpra2 in comparison to 3 wks of age. Two genes, *CDK25B* and *CDKN1B* were specifically up-regulated in erd at 8–10 and 11.9–14.1 wks in comparison to 6.4 wks of age. Notably, there were no changes in expression with age in members of the NDR kinases or Hippo signaling pathway. Details of these results are presented in Additional file 1. All together, these results show that expression changes in the selected genes parallel the structural development of the dog retina in normal and diseased retinas.

#### Disease associated changes in retinal gene expression

**Fig. 4:**
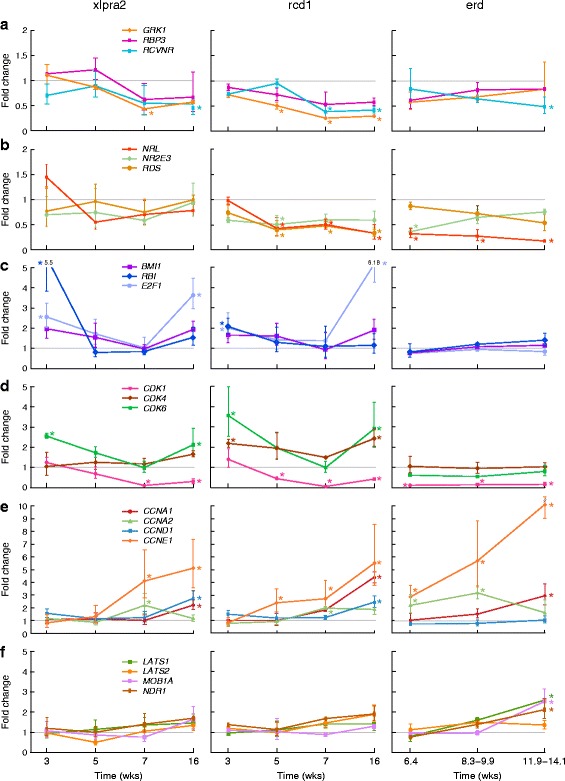
Retinal mRNA expression changes in xlpra2, rcd1, and erd-mutants compared to normal between 3 and 16 wks of age. **a** Photoreceptor function; **b** Photoreceptor development/structure; **c** Cell cycle transcription regulators; **d** Cyclin-dependent kinases; **e** Cyclins; **f** Hippo signaling/NDR kinases. An asterisk indicates statistically significant differences (*p* < 0.05; fold change > +/−2) in expression. Data points are reported as mean +/− SD

To determine the changes in expression as a result of disease, we compared the mutant dogs to age-matched normal controls at different ages. Concurrent with PR cell degeneration, there is decreased expression of genes associated with PR function and structure (Table 1). *GRK1* decreases first in rcd1 at 5 wks, and at 7 wks in xlpra2. By 16 and 11.9–14.1 wks, *RCVRN* is decreased in all 3 diseases. Consistent with incomplete outer segment (OS) formation prior to early PR degeneration in rcd1 dogs, there is decreased expression of *RDS* at 5 wks and subsequent time points. *NRL* and *NR2E3*, genes associated with PR development and cell fate specification [1], are down-regulated in rcd1 at 5, 7 (*NRL* only), and 16 wks, and in xlpra2 also at 16 wks. *NRL*, the upstream regulator of *NR2E3*, is down-regulated at all ages examined in erd, and may account for the formation of hybrid rod/S-cones in the retina following PR cell proliferation [18] (Fig. 4a, b).

**Table 1:** qRT-PCR retinal gene expression results in xlpra2, rcd1, and erd mutants compared to normal retinas

	Ages			
Gene	3 wks	5 wks	7 wks	16 wks
*Cell cycle: cyclins*				
*CCNA1*	n.s	n.s	n.s	+4.4x in rcd1
				+2.2x in xlpra2
				+3.0x in erd (11.9–14.1 w)
*CCNA2*	n.s	n.s	+2.0x in rcd1	n.s
			+2.2x in xlpra2	
			+2.2x in erd (6.4 w)	
			+3.2x in erd (8.3–9.9 w)	
*CCNB1*	n.s	n.s	n.s	+3.3x in rcd1
				+2.8x in xlpra2
*CCND1*	n.s	n.s	n.s	+2.4x in rcd1
				+2.7x in xlpra2
*CCND3*	n.s	n.s	n.s	+2.8x in rcd1
*CCNE1*	n.s	+2.4x in rcd1	+2.7x in rcd1	+5.5x in rcd1
			+4.1x in xlpra2	+5.1x in xlpra2
			+2.8x in erd (6.4 w)	+10x in erd (11.9–14.1 w)
			+5.7x in erd (8.3–9.9 w)	
*Cell cycle: cyclin-dependent kinases*				
*CDK1*	n.s	−2.1x in rcd1	−13.3x in rcd1	−2.2x in rcd1
			−10.2x in xlpra2	−3.4x in xlpra2
			−8.4x in erd (6.4 w)	−6.0x in erd (11.9–14.1 w)
			−6.9x in erd (8.3–9.9 w)	
*CDK2*	+2.7x in rcd1	n.s	n.s	+3.4x in rcd1
*CDK4*	+2.2x in rcd1	n.s	n.s	+2.4x in rcd1
*CDK6*	+3.6x in rcd1	n.s	n.s	+2.9x in rcd1
	+2.5x in xlpra2			+2.1x in xlpra2
*Cell division cycle phosphatases*				
*CDC25A*	n.s	n.s	n.s	n.s
*CDC25B*	n.s	n.s	n.s	n.s
*CDC25C*	n.s	n.s	n.s	+2.1x in rcd1
				+2.7x in xlpra2
*Cyclin-dependent kinase inhibitors*				
*CDKN1A*	n.s	n.s	n.s	n.s
*CDKN1B*	n.s	n.s	n.s	n.s
*CDKN2A*	−3.8x in rcd1	n.s	+3.0x in erd (8.3–9.9 w)	n.s
*Cell cycle transcription regulators*				
*BMI1*	n.s	n.s	n.s	n.s
*E2F1*	+2.0x in rcd1	n.s	n.s	+6.2x in rcd1
	+2.6x in xlpra2			
				+3.7x in xlpra2
*RB1*	+2.1x in rcd1	n.s	n.s	n.s
	+5.5x in xlpra2			
*Hippo signaling/NDR kinases*				
*LATS1*	n.s	n.s	n.s	+2.6x in erd (11.9–14.1 w)
*LATS2*	n.s	n.s	n.s	n.s
*MOB1A*	n.s	n.s	n.s	+2.5x in erd (11.9–14.1 w)
*NDR1*	n.s	n.s	n.s	+2.1x in erd (11.9–14.1 w)
*STK38L (NDR2)* (exons 4–5)	n.s	n.s	No expression in erd	No expression in erd
*STK38L (NDR2)* (exons 6–7)	n.s	n.s	n.s	n.s
*Eye development regulation via proliferation/apoptosis*				
*PAX6*	n.s	n.s	−2.5x in erd (6.4 w)	+2.2x in rcd1
*PCNA*	n.s	n.s	n.s	n.s
*PR function*				
*GRK1*	n.s	−2.0x in rcd1	−3.9x in rcd1	−3.3x in rcd1
			−2.3x in xlpra2	
*RBP3*	n.s	n.s	n.s	n.s
*RCVRN*	n.s	n.s	−2.6x in rcd1	−2.4x in rcd1
				−2.2x in xlpra2
				−2.0x in erd (11.9–14.1 w)
*PR development/structure*				
*CRB1*	n.s	n.s	n.s	n.s
*NRL*	n.s	−2.3x in rcd1	−2.0x in rcd1	−3.0x in rcd1
			−3.2x in erd (6.4 w)	−2.0x in xlpra2
			−3.7x in erd (8.3-9.9 w)	−5.8x in erd (11.9–14.1 w)
*NR2E3*	n.s	−2.0x in rcd1	−2.8x in erd (6.4 w)	n.s
*RDS*	n.s	−2.5x in rcd1	−2.0x in rcd1	−3.0x in rcd1

Statistically significant (*p* < 0.05 and FC > +/−2) differences in retinal gene expression are reported in comparison to normals at different ages (rcd1 and xlpra2: 3, 5, 7, 16 wks; erd: 6.4, 8.3–9.9, 11.9–14.1 wks). The examined genes are reported in alphabetical order within functional groups. The complete list of genes tested is available as Additional file 5. n.s = not statistically significant; + = up-regulated and - = down-regulated compared to normal

Three cell cycle transcription regulators, *BMI1*, *E2F1* and *RB1*, were included in the analysis. While *BMI1* showed no changes associated with disease, both *E2F1* and *RB1* expression was up-regulated in xlpra2 and rcd1 at 3 wks, early in the degenerative process, and *E2F1* at the 16 wk time point. In contrast, there were marked changes in expression of cyclin dependent kinases and cyclins with disease (Table 1). *CDK1* was down-regulated in all diseases beginning at 5 wks in rcd1. *CDKs 2,4,6* were up-regulated at 3 and 16 wks in rcd1, and *CDK6* at the same time points in xlpra2. Up-regulation of *CCNA2* and *CCNE1* occurred in the 3 mutants at 7 wks, and 16 wks (*CCNE1* only); similar up-regulation was found for *CCNA1* at 16 wks. Lastly, *CCNB1*, *D1* and *D3* were up-regulated at 16 wks in rcd1, and *CCNB1* and *D1* in xlpra2 (Fig. 4c-e). These results indicate that changes in the expression of some cyclin dependent kinases and cyclins occur at specific disease stages, and, in some cases, the expression patterns are shared among the 3 diseases.

Disease-associated changes in gene expression for members of the NDR kinases and Hippo pathway were only found in erd at the 11.9–14.1 wks time point. There was up-regulation of *LATS1*, *MOB1A* and *NDR1* (Fig. 4f). There were no expression differences in *STK38L* (*NDR2*), the gene mutated in the disease when primers located 3′ to the deletion were used.

#### Disease-associated changes in RPE gene expression

**Fig. 5:**
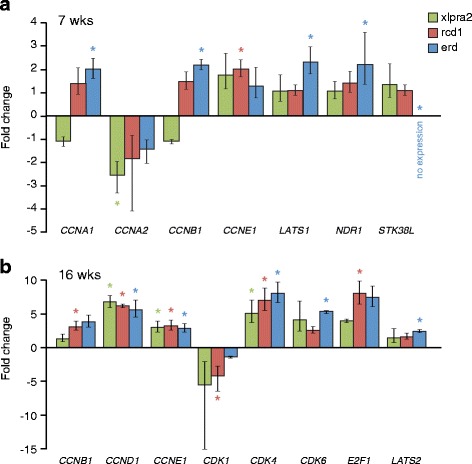
RPE mRNA expression changes in xlpra2, rcd1, and erd-mutants compared to normal at 7 (**a**) and 16 (**b**) wks of age. At 3 wks, there were no differentially expressed genes that reached statistical significance. An asterisk indicates statistically significant differences (*p* < 0.05; fold change > +/−2) in expression. Bars indicate mean +/− SD of triplicate samples

Because of the close RPE-PR physical proximity, shared functional interactions, and the RPE’s role in immune modulation (reviewed by [30], we examined gene expression in the RPE to assess whether the observed retinal expression changes were specific, or also were present in the RPE. To this end, a subset of the genes that were DE in the retinal analyses were used to examine their expression in RPE/choroid samples in normal and mutant retinas at different time points (Additional file 2). Even though there was no evidence of RPE cell mitosis based on PHH3 labeling, we did find that at 16 wks there was a general up-regulation of *CDK4*, *CCND1*, and *CCNE1* in all three diseases. These expression commonalities suggest that similar mechanisms related to the cell cycle pathway might be activated and preserve the integrity in both mutant PRs and RPEs. As well, expression of the cell cycle transcription regulator *E2F1* was increased at 16 wks in rcd1. In general, DE genes in retina and RPE samples were not the same and the ages of dysregulation differed. In contrast, as with the retinal analyses, expression changes in NDR kinases and the Hippo signaling pathway were only found in erd (Fig. 5a, b), although not at the same ages and *LATS2* was specifically DE only in RPE. These results indicate specific and likely independent differences in the expression of cell cycle genes in the 2 different cell types, and do not suggest a key role for RPE cells in retinal degeneration.

### Protein expression and disease

#### Western blot analyses

**Fig. 6:**
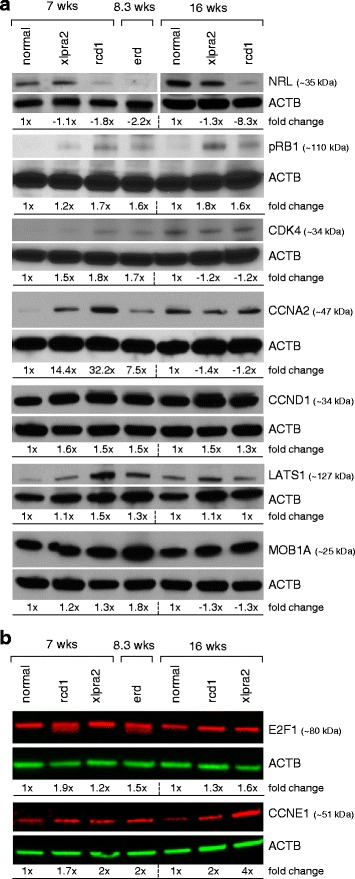
Western blot analysis of normal, xlpra2, rcd1, and erd retinas. **a** Blots exposed on autoradiograph films; **b** Li-COR immunoblotting system. The band intensity normalized to the actin density of the normal control (7 wks for ages 7 and 8.3 wks; 16 wks for 16 wk mutant samples) was set to one, and fold changes were calculated and shown below each of the blots

To complement the mRNA expression studies, we examined a selected number of proteins encoded by genes relevant to the cell cycle and PR cell fate specification, and which were differentially expressed. In addition to NRL, this included transcription regulators (pRB, E2F1), the cyclin dependent kinase CDK4, cyclins (CCNA2, CCND1, CCNE1), and the LATS1 and MOB1A members of the NDR kinase/ Hippo signaling pathway (Fig. 6a, b). The number of genes and time points tested was limited by availability of samples, i.e. one sample/disease/time point, and the lack of specificity to canine tissues for several of the antibodies tested (see Additional file 3).

Relative to normal retina, NRL is down-regulated at 5 (data not shown), 7, and 16 wks in rcd1, and at 8.3 wks in erd, which was the only time point examined by western blot analysis for the latter disease. This result is not unexpected, as the transcription factor NRL is a critical intrinsic regulator of PR development and function, and regulates the expression of several rod-specific genes, including RHO, that, particularly in rcd1, is also down-regulated early in the disease process. The transcription regulators pRB and E2F1 showed similar expression patterns with low levels in normal retinas, and higher levels in the 3 disease models. CDK4 was lower in normals and xlpra2 at 7 wks, while CCND1 and CCNE1 levels were higher in all diseases. The levels of CCNA2 in the 7/8.3 wks diseased retinas were markedly higher. Lastly, we found that expression of MOB1A was specifically elevated in erd, but LATS1 was increased in rcd1 and erd at ~7 wks. In general, there was good agreement between the qRT-PCR and western analyses, which indicate a dysregulation of the expression of some cell cycle proteins in the canine models studied.

#### Immunohistochemistry

**Fig. 7:**
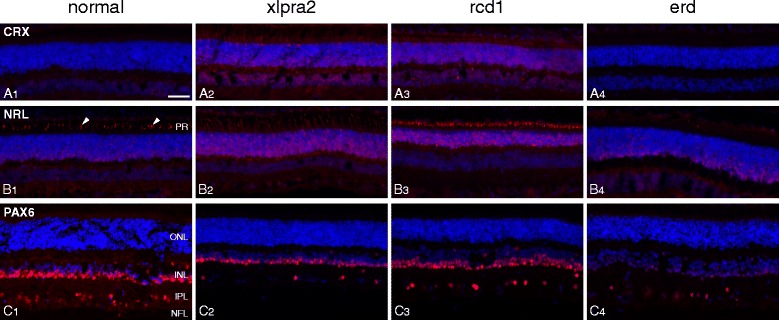
Immunolabeling in normal and mutant retinas for retinal transcription factors. **A** CRX; **B** NRL; **C** PAX6. Samples were from normal (7 wks), xlpra2 (8 wks), rcd1 (7 wks) and erd (CRX-9.1 wks; NRL-7.7 wks; PAX6-14.1 wks). In normal, xlpra2 and rcd1, NRL is expressed in cone outer segments (B1 arrowheads and corresponding locations in B2 and B3) and in the basal aspect of the outer nuclear layer; the rcd1 retina has few rods, and almost no rod outer segments remain so NRL distinctly labels the surviving cone outer segments. PR = photoreceptor, ONL = outer nuclear layer, INL = inner nuclear layer, IPL = inner plexiform layer, NFL = nerve fiber layer. Scale bar = 40 μm

We used IHC to localize the expression of selected proteins, and to qualitatively assess their expression levels. While expression of CRX, a PR-specific transcription factor that plays a role in the differentiation of PR cells, was low in all samples, regardless of disease status, the expression of NRL differed in normal and mutants. Normal retinas showed labeling in the cone outer segments and basal row of ONL nuclei. Similar OS labeling was found in xlpra2 and rcd1, but with a larger area of the ONL occupied by labeled nuclei. In contrast, the erd retina, composed primarily of hybrid rod/S-cones, had a paucity of NRL-labeled cone outer segments, further underlying this particular feature of this disease model. On the other hand, PAX6, a transcription factor present in developing eyes, primarily labeled Müller cell nuclei in normal and mutants, but not PRs (Fig. 7A-C).

**Fig. 8:**
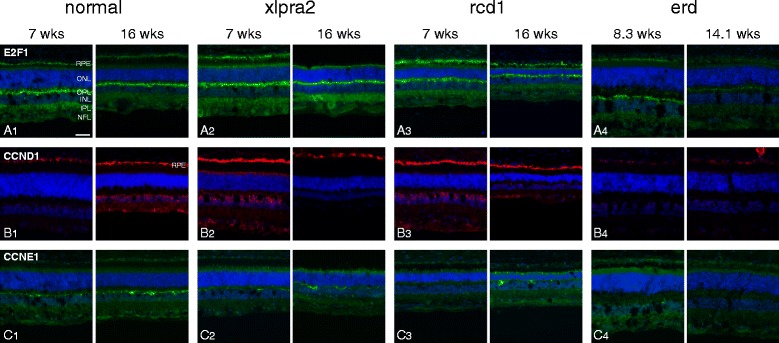
Immunolabeling in normal and mutant retinas for selected cell cycle proteins. Paired sections from normal, xlpra2, rcd1, erd retina taken at two ages were labeled with antibodies against: **A** E2F1, **B** CCND1, **C** CCNE1. More intense labeling of the RPE layer is present in mutants compared with normals with antibodies against E2F1 and CCND1. Note that in the 16 wk xlpra2 retina (B2), the RPE layer has separated from the section during immunolabeling and is not included in the image. RPE = retinal pigment epithelium, ONL = outer nuclear layer, OPL = outer plexiform layer, INL = inner nuclear layer, IPL = inner plexiform layer, NFL = nerve fiber layer. Scale bar = 40 μm

Due to lack of specificity, we were unable to assess immunolabeling with cell cycle specific antibodies against pRB1, CDK1, CDK2, and CCNA2 (Additional file 3). Antibodies against E2F1 showed robust labeling localized to the RPE, IPL and especially the OPL. Mutant retinas, particularly at younger ages, showed more intense RPE labeling (Fig. 8A). Of the 2 cyclins analyzed, CCND1 primarily showed intense labeling over the RPE layer, and, with the exception of erd, was more intense than in controls. In most cases, the labeling was cytoplasmic as well as nuclear (Fig. 8B). In contrast, CCNE1 labeled horizontal processes in the OPL equally in normals and mutants (Fig. 8C).

**Fig. 9:**
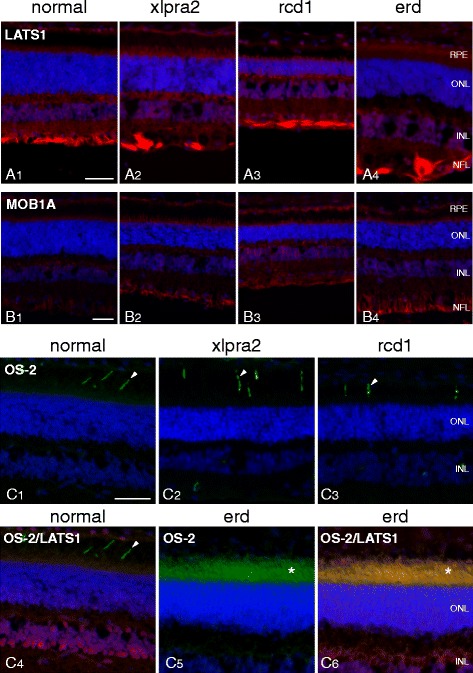
LATS1, MOB1A, OS-2 immunolabeling of normal and mutant retinas. **A** LATS1 labels the INL and GCL/NFL in normal retina. In mutants, there is additional faint labeling of the photoreceptor and RPE layers. **B** Low levels of MOB1A labeling are present in the normal retina, and increase in mutants in the same layers which express LATS1. **C** The OS-2 antibody labels a subpopulation of cones (S-cones; arrowheads) in normal, xlpra2 and rcd1 (C1-3). Dual labeling with OS-2 and LATS1 antibodies in normal shows labeling in different retinal domains (C4). In erd, OS-2 labels the inner and outer segments of the entire photoreceptor population (C5, *), and colocalizes with LATS1 (C6, *). RPE = retinal pigment epithelium, ONL = outer nuclear layer, INL = inner nuclear layer, NFL = nerve fiber layer. Scale bar = 40 μm for (**A**, **B**, **C**)

Immunolocalization of MOB1A was comparable in normal and mutants, and in xlpra2 and rcd1 for LATS1 (Fig. 9A, B). In erd, however, more diffuse labeling of the PR layer was evident with LATS1. With OS-2, a monoclonal antibody that specifically labels S-cones [31], we found labeling of a distinct population of S-cone in normal, xlpra2 and rcd1 (Fig. 9C1–3). In erd, however, the OS-2 antibody resulted in uniform labeling of the entire PR layer (Fig. 9C5). The merged OS-2 and LATS1 images showed no co-localization in normal (Fig. 9C4), but complete co-localization in the PR layer of erd (Fig. 9C6). These results confirm a previous study which described that newly generated PRs in erd are hybrid rod/S-cones [18], and indicates that in erd the LATS1 protein is now expressed in these newly formed cells.

## Discussion

Cell division in normal canine retina ends before 2 wks of age, and PRs mature in the first 5–8 wks after birth [29]. PR proliferation beyond 2 wks occurs infrequently, sporadically, and only in the periphery, adjacent to the ora serrata. In contrast, mitotic PRs are observed in one inherited retinal disease, erd, in which concurrent proliferation and apoptosis of different PR cell populations occurs, and is followed by formation of hybrid rod/S-cones. The cell proliferation and death kinetics are balanced, albeit for a finite period, as the ONL maintains normal thickness until 14 wks of age. Thereafter, the balance tilts towards cell death and the ONL progressively thins [18].

To determine if aberrant PR proliferation is exclusive to erd, we assessed two other phenotypically comparable diseases, xlpra2 and rcd1, and used erd as a mutant control. These diseases are caused by mutations in distinct genes that impair the early phases of PR development, and, generally, have similar structural features and disease time course with rcd1 being faster and more aggressive, followed by xlpra2 and erd [13]. Using PHH3 as a specific marker of the M-phase of the cell cycle, we identified mitotic cells in the ONL in all three diseases, with the greatest number observed in erd. PCNA labeling largely recapitulated trends in PHH3 labeling over time, and the highest numbers of PCNA-positive cells were identified in erd. Confocal microscopy demonstrated conclusively the co-localization of PHH3 with the rod-specific protein RHO, and confirmed that cells undergoing proliferation in all 3 diseases were rod PRs.

The higher overall numbers of PHH3-positive than PCNA-positive cells suggests that the majority of proliferating cells in the ONL are in the M-phase of the cell cycle for all 3 diseases. In xlpra2 and particularly in rcd1, there is marked PR proliferation that is many times higher than that found in normals. In both, proliferation is sustained for the 20 wk period of observation. The magnitude of PR proliferation in the two diseases parallels cell death kinetics based on TUNEL labeling, which is greater in rcd1. Balance between cell proliferation and death in these two diseases does not occur, and the ONL progressively thins beginning at early time points [13]. The overall lack of co-localization of TUNEL- and PHH3-labeled PR cells indicated that different cells undergo proliferation and apoptosis, and that the two events are not linked in a common pathway. Furthermore, the large differences between the number of cells labeled with PHH3 and MAD2, and the smaller number of cells that co-express both markers in the mutant retinas emphasizes that the cells entering the M-phase of the cell cycle do not undergo mitotic checkpoint arrest, but continue to divide, thus contributing new PR cells to the ONL. In contrast, the control retina showed only a very small number of PHH3 positive cells, and all of these co-expressed MAD2.

In *rd1* mice, proliferating cells in the ONL have been reported to be microglia in origin, based on F4/80 immunostaining [14]. In contrast, although the mutant canine retinas showed an increased numbers of CD18-positive microglial cells and processes, these did not co-localize with PHH3 labeling. In addition, GS-positive Müller cell labeling did not co-localize with the dividing PR cells. While the methods used cannot exclude completely a contribution by microglia and/or Müller cells to the dividing cell population in the ONL, the results indicate that such a contribution, if present, is minor. It is possible that some of the PHH3-labeled Müller cells in the vitreal border on the INL in xlpra2 and rcd1 (see Fig. 3C2,3), proliferated and migrated into the ONL. However, prior studies have shown that PAX6 positive Müller glia can express cell cycle markers, but not proliferate [32]. All together, our results show that in the ONL of the 3 canine diseases there are distinct subsets of PR cells one that is dying, while the other is dividing.

A recent study showed that in several rodent models of retinal degeneration there is a reactivation of cell cycle genes before the onset of apoptosis [15]. These findings were in accord with previous studies of neurodegenerative diseases, e.g. Alzheimer disease, Parkinson’s disease and others, where aberrant reentry of susceptible neuronal populations into the cell cycle is associated with the death of post-mitotic, terminally differentiated neurons [33–36]. In the retina of the rodent models, nuclear expression of CDK4, CDK6, and CDK2, as well as phosphorylation of Rb precede PR death. Furthermore, deleting E2F1 in the rd1 model resulted in a transient neuroprotection, whereas deletion of BMI1, the upstream activator of E2F1 and CDKs, resulted in a marked delay in PR loss. At least in the rodent retinas, re-activation of these cell cycle proteins resulted in PR cell death, but not PR mitosis [15].

Unlike these rodent models, PR proliferation is readily observed in 3 early-onset canine models of retinal degeneration (current study and [18]). To begin to assess the mechanistic basis for PR proliferation, we first examined selected genes involved in different phases of the cell cycle. We find that at 3 and 16 wks, the first and last time points examined, mRNA expression of *RB1* and *E2F1* are significantly up-regulated in xlpra2 and rcd1, and protein levels of pRB1 and E2F1 were increased in all 3 diseases. Expressions of *CDK2,4,6* also are up-regulated at the same time points, particularly in rcd1, but *CDK1* expression is down-regulated. Indeed, western analysis of CDK4 showed increased expression at 7 wks in mutants. Furthermore, mRNA expression changes in cyclins were quite uniform across all diseases; *CCNA2* and *CCNE1* were up-regulated at 7 wks, while increased expression of the other cyclins was found at the 16 wk time point. These data were confirmed by western analysis, showing comparable results at the mRNA and protein levels. These results indicate that in the mutant canine retinas there is a dysregulation of cell cycle gene expression in comparison to the normal retina.

Even though the 3 diseases investigated are primary PR degenerations, we also examined the RPE expression changes of selected genes, to determine potential differences or commonalities. We find altered mRNA expression patterns in the RPE at time points when this cell layer is not affected. At 16 wks of age, DE genes were primarily up-regulated, and included *CDK4*, *CCNA1*, and *CCNE1,* which showed increased expression levels in the three diseases. At 7 weeks, on the other hand, statistically higher mRNA levels were observed mainly in erd, and included *NDR1* and *LATS1*, while, as expected, *STK38L* expression of exon 4, deleted in the disease, was absent. Other than *CCNE1*, there was poor correlation between the retina and RPE mRNA expression findings at comparable ages. These results are not unexpected given that the PRs are affected by the disease. Indeed, changes in the RPE occur in response to PR degeneration or alternatively could be due to changes in contaminating tissue, including choroid, microglia/macrophages and white blood cells trapped in the highly vascular choroid. However, in contrast to the retina, the observed differences in RPE cell cycle gene expression in the 3 diseases suggests an activation of pathways that lead to the long-term survival. This hypothesis warrants further studies that could also be performed on primary RPE cell cultures.

While the mRNA and protein expression studies indicate activation of a subset of cell cycle genes in proliferating PR, some of the IHC results are more difficult to reconcile with these findings given the apparent lack of specificity of several of the antibodies. In tissue sections, we could not assess expression of pRB1, CDK1, CDK4, CCNA2, CCNB1, and CCND3. Moreover, in none of the mutant tissues that showed PHH3-labeled proliferating PRs did we find labeling with E2F1, CCND1 or CCNE1 in the ONL, the expected site of these cellular events. Obviously, both qRT-PCR and western analyses include the entire neuroretina, while proteins with IHC were more precisely localized to specific cellular layers, but quantification of labeling intensity is qualitative. These data suggest that the cells expressing different levels of cell cycle-related transcripts may not be the ones that are dividing, thus are not PRs. The specific analysis of these questions will require single cell analysis of PR populations. In contrast to the retina, IHC of the RPE clearly showed expression of several of the cell cycle gene products, e.g. E2F1 and CCND1. Labeling intensity was greater in the mutants vs. controls, and there was a general increase in label intensity in the older affected animals of each genotype. This evidence appears to confirm an activation of specific cell cycle related pathways in RPE that might promote survival of these cells.

Most notably, we find unique changes in the expression of NDR kinases and Hippo signaling genes in erd-mutants, a disease caused by a mutation in the NDR kinase *STK38L* (*NDR2*). We found an up-regulation of the NDR kinases and Hippo signaling pathway members *MOB1A*, *LATS1*, and *NDR1. LATS1* is a tumor suppressor gene expressed in vertebrate retina [37] and stimulates the kinase activity of STK38L upon MOB1A binding and subsequent activation after association with S100B [38, 39]. The increased expression of *LATS1* in erd may be an attempt to suppress the aberrant PR proliferation or to induce apoptosis, as several studies have reported this particular feature of this gene [40–42].

LATS1 and OS-2 co-localization in the PR inner and outer segments was observed with IHC exclusively in erd*,* and confirms the previous finding that aberrant rod/S-cone hybrids are generated following PR proliferation in erd-mutants [18]. Finding marked down-regulation of expression of NRL in erd is also consonant with its known function in PR cell fate specification [1]. *NRL* knockout mouse retinas are almost entirely composed of S-cone PRs. Given that the proliferating population of PRs in the erd retina resulted in L/M-cone and hybrid rod/S-cones, the decreased expression of NRL is not surprising.

The Hippo pathway controls organ size via the regulation of apoptosis and proliferation. It interacts in cell cycle control by CDK1 phosphorylation of YAP, a downstream Hippo pathway effector [43], and cyclin E is a known Hippo pathway target gene [44]. Indeed, the Hippo pathway, via MOB1A activation of LATS1 tumor suppressor can negatively regulate cell proliferation by modulating cyclin A activity [40]. However, as we found that expression changes in *CDK1*, *CCNE1*, *CCNA1*, and *CCNA2* were not specific to erd, but were comparable also in xlpra2 and rcd1, we expect that the observed retinal changes in these genes did not result from the altered Hippo signaling pathway.

## Conclusions

In summary, our results show that in early phases of three retinal degenerative diseases in dogs, PHH3 labeling is present in the outer nuclear layer, an indication that a subset of PR cells have entered the M-phase of the cell cycle. These cells are of rod origin, and there is no evidence that the proliferating cells are microglia or Müller cells. In erd, the proliferating cells transiently maintain the integrity of the ONL in spite of high rates of concurrent apoptotic cell death in cells that are not proliferating (present study; [18]). Similar but lower levels of PR proliferation occur in xlpra2 and rcd1, but the high rate of apoptotic cell death in comparison to PR proliferation result in a more rapid loss of ONL in the early stages of disease. Associated with these events is a dysregulation of cell cycle genes that appears to occur in a different subset of retinal cells, that might play a primary or bystander role in the degenerative process.

## Methods

### Ethics statement

The research was conducted in full compliance with the University of Pennsylvania Institutional Animal Care and Use Committee (IACUC) approval, adhered to the Association for Research in Vision and Ophthalmology (ARVO) Resolution for the Use of Animals in Ophthalmic and Vision Research, and followed the recommendations in the Guide for the Care and Use of Laboratory Animals of the National Institutes of Health. All efforts were made to minimize suffering in the study animals.

### Samples

Retinal and RPE/choroid samples from dogs with a common genetic background, but which segregated with either normal, xlpra2, rcd1, or erd alleles were used. The majority of samples for immunohistochemistry (IHC) and quantitative Real Time-PCR (qRT-PCR) represented archival tissues previously collected for recently published studies [13, 18, 45]; additional samples were collected to complete additional time points. Details of the samples and ages studied are summarized in Additional file 4.

The studied diseases were: 1) X-linked progressive retinal atrophy 2 (xlpra2), an early-onset progressive rod and cone disease with a 2-bp micro-deletion in retinitis pigmentosa GTPase regulator (*RPGR*) exon ORF15 that causes a frameshift and premature stop codon in the translated protein [46]; 2) Rod cone dysplasia 1 (rcd1), an early-onset autosomal recessive rod disease caused by a nonsense mutation in the rod cyclic GMP-phosphodiesterase beta-subunit (*PDE6B*), which results in a stop codon and truncation of the terminal 49 amino acids [24, 47]; and 3) early retinal degeneration (erd), an autosomal recessive disorder of rods and cones caused by an insertion in exon 4 of the serine/threonine kinase 38-like protein (*STK38L*/*NDR2*) [29, 22]. For the present studies, we focused on the most relevant disease-related phases of PR cell death: before cell death peak (*induction*: 3 wks), at cell death peak (*execution*: 5–10 wks), and during sustained but reduced cell death rate (*chronic cell death*: ≥ 14 wks) [13].

### Gene expression quantification

qRT-PCR was performed with strict adherence to the guidelines for minimum information for publication of quantitative real-time PCR experiments (MIQE) [48]. Method details have been published previously [13, 45]. Canine-specific gene amplification was done using either Taqman assays (Applied Biosystems) or primers designed with Primer Express Software v3.0 (Applied Biosystems) using SYBR green (Additional file 5). CT values of each gene were normalized to those of *GAPDH*, the most stable housekeeping gene [13], and comparisons between groups were performed with the ΔΔCT method [49]. Statistical significance of differentially expressed genes (*p* < 0.05; fold change FC > +/−2) was assessed with an unpaired *t*-test. Additional file 4 details the samples (*n* = 3) for each time point and disease.

### Selection of the examined genes

Selection of genes examined in this study (Additional file 5) was mainly based on prior characterization of erd-mutants [18]. As PR proliferation was observed in erd [18], and reactivation of cell cycle proteins accompanies retinal degeneration in rodents [15], we examined retinal expression of a selected number of cell cycle genes belonging to different categories, including cyclins, cyclin-dependent kinases, cell division cycle phosphatases, cyclin-dependent kinase inhibitors, transcription regulators, and selected members of the NDR kinase and Hippo pathway. Genes involved in eye development regulation via proliferation/apoptosis, PR function and development were also evaluated. For the RPE, a smaller subset of non-PR enriched genes was examined. Additional file 5 summarizes the examined genes and their main function.

### Western blot analysis

Equal amounts (60 μg) of total protein as determined by BCA Protein Assay Kit (Thermo Fisher Scientific, Rockford, IL) were separated by 10 % SDS-PAGE under reducing conditions, immunoblotted, and probed with antibodies as previously described [13]. Details on the tested primary antibodies, including their concentrations, are shown in Additional file 3. HRP-conjugated secondary antibodies (Thermo Fisher Scientific) were used at concentrations of 1/10,000 with ACTB as a loading control, and exposed on autoradiograph films (Eastman Kodak, X-oMAT; Rochester, NY). A small subset of proteins was analyzed using the Li-COR Infrared Imaging immunblotting system (Li-COR Biosciences, Lincoln, NE). These were then transferred to nitrocellulose membranes (Li-COR) using the Wet/Tank Blotting System (BioRad) and blocked with Odyssey Blocking Buffer (Li-COR) for 1 h at room temperature. Membranes were then incubated overnight at 4 °C with primary antibodies (Additional file 3) in Odyssey Blocking Buffer containing 0.1 % Tween 20. After primary antibody incubation, membranes were washed with PBS containing 0.1 % Tween 20 (PBST), and incubated for 1 h at room temperature in goat anti rabbit IRDye680RD- and goat anti mouse IRDye800CW-conjugated IgG secondary antibodies (Li-COR) diluted 1/10,000 each in Odyssey Blocking Buffer containing 0.1 % Tween 20. Immunoblots were washed in PBST three times, PBS once, and scanned on Li-COR Odyssey Fc Dual-Mode Imaging System with 700- and 800-nm channels and using the Image Studio Software. Normalization of samples against ACTB was done using Li-COR software for all blots.

### Immunohistochemistry (IHC)

*Standard IHC labeling*: retinal localization of selected proteins (Additional file 3) was performed by IHC using methods previously described [13, 18]. Slides were incubated overnight with one (or two for dual-labeling) primary antibodies, subsequently incubated with fluorochrome-labeled secondary antibodies (Alexa Fluor 568 or 488, Invitrogen, Carlsbad, CA), and cell nuclei stained with DAPI (49,69-diamino-2-phenylindole).

*Low pH Antigen Retrieval (AgRet)*: with E2F1 and MAD2, as well as other not specific or not working antibodies, a low pH antigen retrieval (AgRet) step was used prior to immunolabeling. Tissues were incubated in TBST (1X TBS/0.1 % Tween 20/ 0.02 sodium azide) for 5 min. Fifty milliliters 1x Antigen Unmasking Solution, Low pH (Vector Laboratories Inc, California, USA) was added to a coplin jar containing the tissue slides and transferred to a decloaking chamber (Biocare Medical, Concord, CA, USA) at 125 °C for 90 sec (pressure 25 lb) followed by 90 °C for 10 sec. Tissues were next incubated with 1X TBST for 10 min and washed with 1X PBS for 5 min each. Immunolabeling was then performed as noted above for standard IHC labeling.

*PHH3 and PCNA single and dual labeling:* to identify outer nuclear layer (ONL) cells that undergo mitosis, we used the PHH3 antibody that distinguishes mitotic cells from apoptotic cells and karyorrhectic debris [50]. The proliferative cell nuclear antigen (PCNA) antibody was used to identify cells that are proliferating and/or undergoing DNA repair [26], while the MAD2 antibody was used to identify those cells in mitotic checkpoint arrest [51]. Finally, to detect apoptotic ONL cells we used the TUNEL (terminal deoxynucleotidyl transferase mediated biotinylated UTP nick end labeling) in situ cell death detection kit (Roche Applied Science, Indianapolis, IN).

To analyze PHH3 and PCNA single and dual labeling, we used the standard IHC protocol [13, 18] with the following modifications. Prior to the initial treatment with 1x PBS/0.25 % triton X-100, single (PHH3, PCNA) or dual-labeled (PHH3 with CD18, rod opsin, TUNEL, and Glutamine Synthetase - GS) sections were boiled in coplin jars containing high pH antigen unmasking solution (Vector Laboratories) in a 600 watt microwave at 30 % power for 10 min, cooled at room temperature for 30 min, and washed 2x with PBS. The samples were then processed using standard IHC methods for dual labeling.

TUNEL and PHH3 dual-labeled sections were cooled for 45 min, blocked with 10 % goat serum, incubated in 0.1 % Triton X-100/0.1 % Na citrate permeabilization solution, and washed 2x in PBS. A total of 50 μL TUNEL reaction mixture was added for 1 h at 37 °C in the dark, while negative controls only received 50 μL label solution. Slides were then rinsed 3x with PBS and incubated with the PHH3 antibody as described above. For PHH3/MAD2 dual labeling, the previously described low pH AgRet method was used, with an additional dual labeling step for MAD2.

Positive cells in the ONL were counted in triplicate under fluorescent microscopy for the entirety of each retina section. The number of positive cells was then normalized to the calculated area of the ONL using a conventional hematoxylin and eosin (H&E) stained image from a serial section congruent with the fluorescently labeled slide. Details of the cell counting and analysis methods are shown in [13, 18].

*IHC Imaging:* The Gelvatol (pH = 8.5; Sigma-Aldrich)-mounted slides were examined with an epifluorescence microscope (Axioplan; Carl Zeiss Meditec, Thornwood, NY). Epifluorescence or transmitted light images were captured with a Spot 4.0 camera (Diagnostic Instruments Inc., Sterling Heights, MI) and displayed with Photoshop and Illustrator (Adobe, San Jose, CA). In addition, dual-labeled slides with PHH3 and rod opsin antibodies were visualized with a Leica TCS SP5 II scanning laser confocal microscope (Leica Microsystems, Wetzlar, Germany) with a resonant scanner for single cell antibody co-localization analysis. For each antibody, control and affected dogs were processed at the same time with the same antibody concentration and lot number; when capturing images for documentation, the same magnification and camera settings were used in all the samples. For those proteins involved in cell cycle, tonsil and thymus tissues from an 11.7 wk old normal dog were used to confirm specificity of immunolabeling.

### Availability of supporting data

The datasets supporting the conclusions of this article are included within the article and its additional files.

## Additional files

Additional file 1:
**qRT-PCR retinal gene expression results during development in normal, xlpra2, rcd1, and erd.** Statistically significant (*p* < 0.05 and FC > +/−2) gene expression differences are reported between different developmental ages (within group comparison) in each of the normal, rcd1, xlpra2, and erd groups. (DOCX 30 kb) Additional file 2:
**qRT-PCR expression results in the RPE of xlpra2, rcd1, and erd mutants compared to normal.** Statistically significant (*p* < 0.05 and FC > +/−2) gene expression differences are reported in each of the rcd1, xlpra2, and erd groups compared with normals at different ages (3, 7, and 16 wks). (DOCX 18 kb) Additional file 3:
**List of antibodies used for immunohistochemistry (IHC) and western blot (WB).** Antibodies are reported with the symbol of the corresponding protein, source (commercial company name and catalogue number or the name of the person who provided it), description of host and antibody type (polyclonal or monoclonal), concentrations used for either IHC or WB and the expected size of the protein in kDa. (DOCX 16 kb) Additional file 4:
**Summary of diseases and ages tested in the different study procedures.** The table summarizes the number and ages of the dogs tested for each tissue type in the different procedures (i.e. qRT-PCR, western blot analysis, and IHC/morphology). (DOCX 16 kb) Additional file 5:
**Genes tested by qRT-PCR in retina and RPE.** The complete list of all the genes examined by qRT-PCR is reported, with gene symbols and alternative symbols, gene names, main functions, and TaqMan assay numbers (ABI; http://www3.appliedbiosystems.com/AB_Home/index.htm) or primer sequences. (DOCX 22 kb)

## Abbreviations

ᅟ: The gene symbols and corresponding gene descriptions of all the genes tested are shown in Additional file 5.
ABI: Applied Biosystems
AgRet: antigen retrieval
ARVO: association for research in vision and ophthalmology
BH: Benjamini Hochberg
Da: Dalton
DAPI: 49,69-diamino-2-phenylindole
DE: differentially expressed
erd: early retina degeneration
FC: fold change
FFB: Foundation Fighting Blindness
GAPDH: glyceraldehyde 3-phosphate dehydrogenase
GCL: ganglion cell layer
GS: Glutamine Synthetase
H&E: hematoxylin and eosin
IACUC: Institutional Animal Care and Use Committee
IHC: immunohistochemistry
INL: inner nuclear layer
IPL: inner plexiform layer
IS: inner segment
MAD2: Mitotic Arrest Deficient-2
MIAME: minimum information about a microarray experiment
MIQE: minimum information for publication of quantitative real-time PCR experiments
n.s: not statistically significant
n.t: not tested
NFL: nerve fiber layer
ONL: outer nuclear layer
OPL: outer plexiform layer
OS: outer segment
PCNA: proliferative cell nuclear antigen
PDE6B: rod cyclic GMP phosphodiesterase 6 ß subunit
PHH3: phospho histone H3
PR: photoreceptor
qRT-PCR: quantitative real-time PCR
rcd1: rod cone dysplasia 1
RPE: retinal pigment epithelium
RPGR: retinitis pigmentosa GTPase regulator
SD: standard deviation
STK38L: serine/threonine kinase 38 like
TUNEL: terminal deoxynucleotidyl transferase mediated biotinylated UTP nick end labeling
WB: western blot
wks: weeks
xlpra2: X-linked progressive retinal atrophy 2
XLRP: X-linked retinitis pigmentosa
