# Supplementary material for: Photoreceptor proliferation and dysregulation of cell cycle genes in early onset inherited retinal degenerations
Source: BMC Genomics. 2016 Mar 11;17:221. doi: 10.1186/s12864-016-2477-9 (PMC4788844; doi:10.1186/s12864-016-2477-9)
Supplement: Additional file 1: — qRT-PCR retinal gene expression results during development in normal, xlpra2, rcd1, and erd. Statistically significant (p < 0.05 and FC > +/−2) gene expression differences are reported between different developmental ages (within group comparison) in each of the normal, rcd1, xlpra2, and erd groups. (DOCX 30 kb) [file 12864_2016_2477_MOESM1_ESM.docx]

**Additional file 1. qRT-PCR retinal gene expression results during development in normal, xlpra2, rcd1, and erd retinas.** Statistically significant (p<0.05 and FC>+/-2) differences are reported between different developmental ages (within group comparison) in normal, rcd1 and xlpra2 (5 vs. 3, 7 vs. 3, 16 vs. 3, 7 vs. 5, 16 vs. 5, and 16 vs. 7 wks), and erd (8.3/9.9 vs. 6.4, 11.9/14.1 vs. 6.4, and 11.9/14.1 vs. 8.3/9.9) retinas. The examined genes are listed in alphabetical order within functional groups. The complete list of genes tested is available as Additional file 5.

* = in erd-mutants the gene *STK38L* (exons 4-5) was not expressed; n.s = not statistically significant; + = up-regulated and - = down-regulated at the older age for each group of comparisons.

| **Gene** | **Age comparisons** | | | | | |
| --- | --- | --- | --- | --- | --- | --- |
| **normal, rcd1, xlpra2:** | **5-3 wks** | **7-3 wks** | **16-3 wks** | **7-5 wks** | **16-5 wks** | **16-7 wks** |
| **erd:** |  |  |  | **8/10-6.4 wks** | **12/14-6.4 wks** | **12/14-8/10 wks** |
| ***Cell cycle: cyclins*** | | |  |  |  |  |
| *CCNA1* | n.s | n.s | -3.2x normal | n.s | -2.2x normal | n.s |
| *CCNA2* | n.s | n.s | n.s | n.s | n.s | n.s |
| *CCNB1* | n.s | -2.2x normal | -3.8x normal | +3.2x erd | n.s | n.s |
| *CCND1* | n.s | n.s | n.s | n.s | n.s | n.s |
| *CCND3* | n.s | -2.6x xlpra2 | n.s | n.s | n.s | +2.8x rcd1  -2.8x erd |
| *CCNE1* | n.s | -3.0x normal | -4.7x normal | n.s | -3.0x normal  +2.3x erd | n.s |
| ***Cell cycle: cyclin-dependent kinases*** | | |  |  |  |  |
| *CDK1* | n.s | +15.4x normal | +8.3x normal  +2.6x rcd1  +2.0x xlpra2 | +8.5x normal | +4.6x normal  +4.3x rcd1 | +3.2x rcd1 |
| *CDK2* | n.s | -2.2x rcd1 | n.s | n.s | n.s | +2.5x rcd1 |
| *CDK4* | n.s | -2.2x rcd1 | n.s | n.s | n.s | n.s |
| *CDK6* | n.s | -2.4x rcd1 | n.s | n.s | n.s | n.s |
| ***Cell division cycle phosphatases*** | | |  |  |  |  |
| *CDC25A* | n.s | n.s | n.s | n.s | n.s | n.s |
| *CDC25B* | n.s | n.s | n.s | +4.4x erd | +2.5x erd | n.s |
| *CDC25C* | n.s | n.s | -2.9x rcd1 | n.s | -2.1x rcd1 | n.s |
| ***Cyclin-dependent kinase inhibitors*** | | |  |  |  |  |
| *CDKN1A* | +2.1 rcd1 | n.s | n.s | n.s | n.s | n.s |
| *CDKN1B* | n.s | n.s | -2.2x normal | +2.1x erd | +2.2x erd | n.s |
| *CDKN2A* | -3.7x normal | n.s | n.s | +2.5x rcd1 | n.s | n.s |
| ***Cell cycle transcription regulators*** | |  |  |  |  |  |
| *BMI1* | n.s | n.s | n.s | n.s | n.s | n.s |
| *E2F1* | n.s | -2.4x rcd1  -4.0x xlpra2 | -3.0x normal  -2.1x xlpra2 | -2.3x xlpra2 | -2.6x normal | +2.5x rcd1 |
| *RB1* | -4.4x xlpra2 | -5.4x xlpra2 | -2.2x rcd1  -4.3x xlpra2 | n.s | -2.2x rcd1 | n.s |
| ***Hippo signaling/NDR kinases*** | |  |  |  |  |  |
| *LATS1* | n.s | n.s | n.s | n.s | n.s | n.s |
| *LATS2* | n.s | n.s | n.s | n.s | n.s | n.s |
| *MOB1A* | n.s | n.s | n.s | n.s | n.s | n.s |
| *NDR1* | n.s | n.s | n.s | n.s | n.s | n.s |
| *STK38L*  (exons 4-5) | n.s | n.s | n.s | n.s* | n.s* | n.s* |
| *STK38L*  (exons 6-7) | n.s | n.s | n.s | n.s | n.s | n.s |
| ***Eye development regulation via proliferation/apoptosis*** | | | |  |  |  |
| *PAX6* | -2.1x rcd1 | n.s | n.s | +2.9x rcd1  +2.2x erd | +2.7x rcd1 | n.s |
| *PCNA* | n.s | -2.3x rcd1 | -2.3x normal  -2.6x rcd1 | n.s | n.s | n.s |
| ***PR function*** |  |  |  |  |  |  |
| *GRK1* | n.s | n.s | n.s | n.s | n.s | n.s |
| *RBP3* | n.s | n.s | n.s | n.s | n.s | n.s |
| *RCVRN* | n.s | -2.3x rcd1 | -2.4x rcd1 | n.s | n.s | n.s |
| ***PR development/structure*** | |  |  |  |  |  |
| *CRB1* | n.s | n.s | n.s | n.s | n.s | n.s |
| *NRL* | n.s | n.s | n.s | n.s | n.s | n.s |
| *NR2E3* | n.s | -2.1x normal  -2.1x rcd1  -2.5x xlpra2 | -3.2x normal  -3.1x rcd1  -2.3x xlpra2 | n.s | -2.1x normal | n.s |
| *RDS* | n.s | n.s | -2.7x rcd1 | n.s | n.s | n.s |
