# Supplementary material for: Photoreceptor proliferation and dysregulation of cell cycle genes in early onset inherited retinal degenerations
Source: BMC Genomics. 2016 Mar 11;17:221. doi: 10.1186/s12864-016-2477-9 (PMC4788844; doi:10.1186/s12864-016-2477-9)
Supplement: Additional file 2: — qRT-PCR expression results in the RPE of xlpra2, rcd1, and erd mutants compared to normal. Statistically significant (p < 0.05 and FC > +/−2) gene expression differences are reported in each of the rcd1, xlpra2, and erd groups compared with normals at different ages (3, 7, and 16 wks). (DOCX 18 kb) [file 12864_2016_2477_MOESM2_ESM.docx]

**Additional file 2.** **qRT-PCR expression results in the RPE of xlpra2, rcd1, and erd mutants compared to normal.** Statistically significant (p<0.05; FC>+/-2) differences are reported in comparison to normals at different ages (3, 7, and 16 wks). The examined genes are reported in alphabetical order within functional groups. The complete list of genes tested is available as Additional file 5. n.s = not statistically significant; n.t = not tested; + = up-regulated and - = down-regulated compared to normal.

|  |  | | **Ages** | |  | |
| --- | --- | --- | --- | --- | --- | --- |
| **Gene** | **3 wks** | | **7 wks** | | **16 wks** | |
| ***Cell cycle: cyclins*** | |  | |  | |  |
| *CCNA1* | n.t | | +2.0x in erd | | n.s | |
| *CCNA2* | n.t | | -2.5x in xlpra2 | | n.s | |
| *CCNB1* | n.t | | +2.2x in erd | | +3.1x in rcd1 | |
| *CCND1* | n.s | | n.s | | +6.2x in rcd1  +6.7x in xlpra2  +5.6x in erd | |
| *CCNE1* | n.s | | +2.0x in rcd1 | | +3.2x in rcd1  +2.9x in xlpra2  +2.8x in erd | |
| ***Cell cycle: cyclin-dependent kinases*** | |  | |  | |  |
| *CDK1* | n.s | | n.s | | -4.3x in rcd1 | |
| *CDK4* | n.s | | n.s | | +6.9x in rcd1  +5.0x in xlpra2  +8.0x in erd | |
| *CDK6* | n.s | | n.s | | +5.2x in erd | |
| ***Cell cycle transcription regulators*** | |  | |  | |  |
| *E2F1* | n.s | | n.s | | +7.9x in rcd1 | |
| *RB1* | n.s | | n.s | | n.s | |
| ***Hippo signaling/NDR kinases*** | |  | |  | |  |
| *LATS1* | n.t | | +2.3x in erd | | n.s | |
| *LATS2* | n.t | | n.s | | +2.4x in erd | |
| *MOB1A* | n.t | | n.s | | n.s | |
| *NDR1* | n.t | | +2.2x in erd | | n.s | |
| *STK38L (NDR2)*  (exons 4-5) | n.t | | No expression in erd | | No expression in erd | |
| *STK38L (NDR2)*  (exons 6-7) | n.t | | n.s | | n.s | |
| ***Eye development regulation via proliferation/apoptosis*** | | | |  | |  |
| *PAX6* | n.t | | n.s | | n.s | |
