# Supplementary material for: Photoreceptor proliferation and dysregulation of cell cycle genes in early onset inherited retinal degenerations
Source: BMC Genomics. 2016 Mar 11;17:221. doi: 10.1186/s12864-016-2477-9 (PMC4788844; doi:10.1186/s12864-016-2477-9)
Supplement: Additional file 3: — List of antibodies used for immunohistochemistry (IHC) and western blot (WB). Antibodies are reported with the symbol of the corresponding protein, source (commercial company name and catalogue number or the name of the person who provided it), description of host and antibody type (polyclonal or monoclonal), concentrations used for either IHC or WB and the expected size of the protein in kDa. (DOCX 16 kb) [file 12864_2016_2477_MOESM3_ESM.docx]

**Additional file 3.** **List of antibodies used for immunohistochemistry (IHC) and western blot (WB).** Antibodies are reported with the symbol of the corresponding protein, source including commercial company name (catalogue number) or the name of the person who provided it, description, and concentrations used for either IHC or WB (with the expected size in kDa).

| **Protein** | **Source or company**  **(catalog number)*** | **Description** | **IHC**  **concentration** | **WB concentration** | **Expected size (kDa)** |
| --- | --- | --- | --- | --- | --- |
| ACTB | Millipore (MAB1501) | Mouse monoclonal | - | 1/10,000 | 43 |
| CD18 | Provided by P. Moore  (clone CA16.3C10) | Mouse monoclonal | 1/10 | - |  |
| CCNA2 | Aviva (ARP30159_P050) | Rabbit polyclonal | Not specific | 1/500 | 47 |
| CCNB1 | Cell Signaling (12231P) | Rabbit monoclonal | Not specific | Did not work | 58 |
| CCND1 | Aviva (ARP30163_P050) | Rabbit polyclonal | 1/500 | 1/500 | 34 |
|  | Aviva (ARP33370_P050) | Rabbit polyclonal | Did not work | Did not work | 34 |
| CCND3 | Santa Cruz (sc-182) | Rabbit polyclonal | Not specific | Did not work | 33 |
| CCNE1 | Hypromatrix (HM1105) | Rabbit polyclonal | Did not work | 1/1,000 | 51 |
| CDK1 | Hypromatrix (HM1083) | Mouse monoclonal | Not specific | Did not work | 34 |
| CDK4 (C-22) | Santa Cruz (sc-260) | Rabbit polyclonal | Not specific | 1/500 | 34 |
| CRX | Provided by A. Swaroop | Rabbit polyclonal | 1/100 | Did not work | 37 |
| E2F1 (C-20) | Santa Cruz (sc-193) | Rabbit polyclonal | 1/1,000 | 1/1,000 | 60 (actual 80) |
| GS | Chemicon (MAB302) | Mouse monoclonal | 1/1,000 | - |  |
| LATS1 | Provided by D. Farber | Rabbit polyclonal | 1/1,000 | 1/800 | 127 |
| MOB1A | Aviva (ARP65686_P050) | Rabbit polyclonal | 1/100 | 1/500 | 25 |
| MAD2 | BD Biosciences (610678) | Mouse monoclonal | 1/1,000 | - |  |
| NRL | Santa Cruz Biotechnology (sc-33183) | Rabbit polyclonal | - | 1/200 | 26 |
|  | Provided by A. Swaroop | Rabbit polyclonal | 1/100 | - |  |
| OS-2 | Provided by A. Szel | Mouse monoclonal | 1/100 | - |  |
| PAX6 | Covance (PRB-278P) | Rabbit polyclonal | 1/1,000 | 1/800 | 47-50 |
| PCNA | Millipore (MAB424) | Mouse monoclonal | 1/100 | - |  |
| PHH3 | Millipore (06-570) | Rabbit polyclonal | 1/500 | - |  |
| pRB1 | Cell signaling (9308) | Rabbit polyclonal | Not specific | 1/1,000 | 110 |
| RHO | Millipore (MAB5316) | Mouse monoclonal | 1/1,000 | - |  |

*: commercial sources for antibodies are Aviva Systems Biology, San Diego, CA; BD Biosciences, San Jose, CA; Cell Signaling, Danvers, MA; Chemicon, Temecula, CA; Covance, Princeton, NJ; Hypromatrix, Worcester, MA; Millipore, Billerica, MA; Santa Cruz Biotechnology, Santa Cruz, CA.
