# Supplementary material for: Photoreceptor proliferation and dysregulation of cell cycle genes in early onset inherited retinal degenerations
Source: BMC Genomics. 2016 Mar 11;17:221. doi: 10.1186/s12864-016-2477-9 (PMC4788844; doi:10.1186/s12864-016-2477-9)
Supplement: Additional file 4: — Summary of diseases and ages tested in the different study procedures. The table summarizes the number and ages of the dogs tested for each tissue type in the different procedures (i.e. qRT-PCR, western blot analysis, and IHC/morphology). (DOCX 16 kb) [file 12864_2016_2477_MOESM4_ESM.docx]

**Additional file 4. Summary of diseases and ages tested in the different study procedures.**

| **Status** | | **Procedures: age in wks (number of dogs)** | | |  |
| --- | --- | --- | --- | --- | --- |
|  | **qRT-PCR** | | **Western blot analysis** | **IHC/morphology** | |
| normal | Retina: 3 (3), 5 (3), 7 (3), 16 (3)  RPE: 3 (3), 7 (3), 16 (3)  Tonsil, thymus: | | 5 (1), 7 (1), 16 (1) | 2 (1), 4 (1), 6 (1), 7 (1), 9 (1), 11.7 (1), 12 (1), 16 (1)  11.7 (1) | |
| xlpra2 | Retina: 3 (3), 5 (3), 7 (3), 16 (3)  RPE: 3 (3), 7 (3), 16 (3) | | 7 (1), 16 (1) | 4 (1), 6.7 (1), 8 (1), 12 (1), 16 (1), 20 (1) | |
| rcd1 | Retina: 3 (3), 5 (3), 7 (3), 16 (3)  RPE: 3 (3), 7 (3), 16 (3) | | 5 (1), 7 (1), 16 (1) | 2 (1), 4 (1), 5 (1), 6 (1), 7 (1), 12 (1), 16 (1), 20 (1) | |
| erd | Retina: 6.4 (2), 8.3-9.9 (3), 11.9-14.1 (2)  RPE: 7 (3), 12.3-15.2 (2) | | 8.3 (1) | 4.3 (1), 7.7 (1), 7.8 (1), 8.3 (1), 9.1 (1), 11.6 (2), 12.3 (1), 14.1 (1) | |
