# Supplementary material for: Photoreceptor proliferation and dysregulation of cell cycle genes in early onset inherited retinal degenerations
Source: BMC Genomics. 2016 Mar 11;17:221. doi: 10.1186/s12864-016-2477-9 (PMC4788844; doi:10.1186/s12864-016-2477-9)
Supplement: Additional file 5: — Genes tested by qRT-PCR in retina and RPE. The complete list of all the genes examined by qRT-PCR is reported, with gene symbols and alternative symbols, gene names, main functions, and TaqMan assay numbers (ABI; http://www3.appliedbiosystems.com/AB_Home/index.htm) or primer sequences. (DOCX 22 kb) [file 12864_2016_2477_MOESM5_ESM.docx]

**Additional file 5. Genes tested by qRT-PCR in retina and RPE*.** Genes are reported with their symbols (in parenthesis alternative symbols), descriptions, main functions, and TaqMan assay numbers (ABI; http://www3.appliedbiosystems.com/AB_Home/index.htm) or primer sequences.

| **Gene symbol**  **(alternative symbol)** | **Gene description** | **Main function**** | **TaqMan assay or primer sequences** |
| --- | --- | --- | --- |
| *GAPDH****** | Glyceraldehyde 3-phosphate dehydrogenase | Housekeeping | Hs02786624_g1 |
| *BMI1* | BMI1 polycomb ring finger oncogene | Maintains the transcriptionally repressive state of genes, involved in PR degeneration in mice and rats. | **F**: TTTTCCGGGATATTTTATCAAGCA  **R**: GGGATTTAGCTCAGTGATCTTGATTC |
| *CCNA1****** | Cyclin A1 | Cell cycle and proliferation (controls and promotes G1/S (start) and G2/M (mitosis) transitions). | Cf02633425_m1 |
| *CCNA2****** (*CCNA/CCN1*) | Cyclin A2 | Cell cycle and proliferation (controls and promotes G1/S (start) and G2/M (mitosis) transitions). | Cf02695410_m1 |
| *CCNB1****** | Сyclin B1; G2/mitotic-specific cyclin B1 | Cell cycle and proliferation (controls the G2/M transition). | **F**: GCCCTCTACCCCTGCATTTC  **R**: TGCTCAACATCAACCTCTCCAA |
| *CCND1****** (*BCL1/PRAD1*) | Cyclin D1; B-cell CLL/lymphoma 1 | Cell cycle and proliferation (regulatory subunit of CDK4 or CDK6, whose activity is required for G1/S transition). | **F**: CATCTACACTGACAACTCCATCC  **R**: CAGGTTCCACTTCAGTTTGTTC |
| *CCND3* | Cyclin D3, G1/S-specific cyclin D3 | Cell cycle and proliferation (regulatory subunit of CDK4 or CDK6, whose activity is required for G1/S transition). | **F**: GTGTGTGCAGAGGGAGATCAAG  **R**: CACAGACCTCCAGCATCCAGTA |
| *CCNE1****** | Cyclin E1 | Cell cycle and proliferation (G1/S-specific). | **F**: TGGCCCCAGTTTTTGCA  **R**: CCTCTCGCAGTCCTGTCAATTT |
| *CDC25A* | Cell division cycle 25A | Phosphatase required for progression from G1 to S phase of the cell cycle, dephosphorylates CDK1 and thus activates it. | **F**: AACCTTGGCAATCGATGCA  **R**: ACCGGGCGATGGAGCTA |
| *CDC25B* | Cell division cycle 25B | Phosphatase required for G2/M phases of the cell cycle progression, functions as a dosage-dependent inducer of mitotic progression, dephosphorylates CDK1 and thus activates it. | **F**: GAGCAGGCCATCCAAGCA  **R**: GAAGCGTCGAATGGCAAACT |
| *CDC25C* | Cell division cycle 25C | Phosphatase required for progression of the cell cycle, functions as a dosage-dependent inducer of mitotic progression, when phosphorylated activates G2 cells into prophase, dephosphorylates CDK1 and thus activates it. | **F**: TCTGCCAACCTAAGCGTTTTG  **R**: GATTCGAAAGATCAAGGCAACATT |
| *CDK1****** (*CDC2*) | Cyclin-dependent kinase 1, Cell division control protein 2 homolog | Cell cycle and proliferation (essential for G1/S and G2/M transitions). | **F**: ACTCTTCAGAATTTTCAGAGCTTTGG  **R**: GATTCCACTTCTGGCCACACTT |
| *CDK2* | Cyclin-dependent kinase 2 | Cell cycle and proliferation (activity restricted and essential for G1/S transition, involved in PR degeneration in mice and rats). | **F**: CCTCATCAAGAGCTATCTGTTCCA  **R**: CCCGATGAGAATGGCAGAAG |
| *CDK4****** | Cyclin-dependent kinase 4 | Cell cycle and proliferation (activity restricted and essential for G1/S transition, phosphorylates RB1, involved in PR degeneration in mice and rats). | **F**: CAGTGGAGACCATCAAGGATCTG  **R**: GCAGTTGGCATGAAGGAAATCT |
| *CDK6****** | Cyclin-dependent kinase 6 | Cell cycle and proliferation (essential for G1/S transition, involved in PR degeneration in mice and rats. | **F**: CAGTGGTCGTCACACTGTGGTA  **R**: GGTGGCATAGCTGGACTGAAG |
| *CDKN1A* (*P21, CIP1*) | Cyclin-dependent kinase inhibitor 1A | Potent cyclin-dependent kinase inhibitor, binds to and inhibits activity of cyclin-CDK2 or -CDK4 complexes, regulator of cell cycle progression at G1. | **F**: CACCTCTCAGGGCCGAAAA  **R**: GGCGTTTGGAGTGATAGAAATCTG |
| *CDKN1B* (*P27, KIP1*) | Cyclin-dependent kinase inhibitor 1B | Cyclin-dependent kinase inhibitor, binds to and prevents activation of CCNE-CDK2 or CCND-CDK4 complexes, controls cell cycle progression at G1. | **F**: CCGACGATTCCTCTCCTCAA  **R**: GGAACCGTCTGAGACGTTTTCT |
| *CDKN2A* (*P16*, *INK4A*) | Cyclin-dependent kinase inhibitor 2A | Cyclin-dependent kinase inhibitor, induces cell cycle arrest in G1 and G2 phases, acts as a tumor suppressor, induces G2 arrest and apoptosis in a p53-independent manner by preventing activation of cyclin B1/CDC2 complexes. | **F**: TACGGAAGGTGCGGAAGGTC  **R**: TGAAAAAGGAGTGCTCTGGGC |
| *CRB1* | Crumbs homolog 1 (Drosophila) | Localizes to the inner segment of mammalian PRs, plays a role in PR morphogenesis. | **F**: CCTACGAAGGCCCGAACTG  **R**: AACTCTTGTCAAGATTAAAAGCAGCAA |
| *E2F1****** | E2F transcription factor 1, Retinoblastoma-associated protein 1 | Cell cycle and proliferation (can mediate both cell proliferation and p53-dependent/independent apoptosis, involved in PR degeneration in mice and rats. | **F**: CTCCAAGCCATAGACTCCTCAGA  **R**: GGGCACAGGAAAACGTCAAT |
| *GRK1* (*RHOK/GPRK1/RK*) | G protein-coupled receptor kinase 1, rhodopsin kinase | Phosphorylates rhodopsin to initiate its deactivation. | **F**: AGGGTGCTATGGTGGAGAAGAA  **R**: GTTTCAAATGCATAGGCCAGAGA |
| *LATS1****** (*WARTS/wts*) | LATS, large tumor suppressor, homolog 1 (Drosophila) | Hippo pathway, tumor suppressor gene. | Cf02626754_m1 |
| *LATS2****** | LATS, large tumor suppressor, homolog 2 (Drosophila) | Hippo pathway, tumor suppressor gene. | Cf02633924_m1 |
| *MOB1A****** (*MOBKL1A/MOB1B/MATS2*) | MOB kinase activator 1A | Hippo pathway, activates LATS1 and LATS2. | Cf02649192_m1 |
| *NDR1****** (*STK38/NDR*) | Serine/threonine kinase 38 | Regulates cell cycle, proliferation, apoptosis. | **F**: CAACCTTCTCCTGGACAGCAA  **R**: CTGTGCAAAGGCCGAAGTC |
| *NRL* (*RP27)* | Neural retina leucine zipper | Regulates PR development | Cf02655725_m1 |
| *NR2E3* (*ESCS/PNR/RNR/rd7*) | Nuclear receptor subfamily 2, group E, member 3 | Proliferation; activates rod and represses cone development. | **F**: TGAAGGCCCTGGTCCTCTT  **R**: ACGTGCTCAGGATCCTTCAG  **TaqMan:** CACACCAGAAACTCG |
| *PAX6****** (*AN/AN2/MGDA/WAGR*) | Paired homeobox gene 6 | Regulates eye development. | Cf02649657_g1 |
| *PCNA* | Proliferating cell nuclear antigen | Cell proliferation. | Cf02634115_m1 |
| *RB1****** | retinoblastoma 1, osteosarcoma | Negative regulator of cell cycle and proliferation. | **F**: TTATCGAGTAATGGAATCCATGCTTA  **R**: TGTCGTCATTCAGGAGTTTGCT |
| *RBP3* (*IRBP*) | Retinol binding protein 3, interstitial, interphotoreceptor retinoid-binding protein | Shuttles retinoids in PRs. | Cf02676369_m1 |
| *RCVRN* (*RCV1*) | Recoverin, cancer-associated retinopathy protein | Inhibits rhodopsin kinase, regulates sensory adaptation in retina. | Cf02642703_m1 |
| *RDS* (*PRPH2/CACD2/DS/RP7/rd2*) | Peripherin 2 (retinal degeneration, slow) | Prevents PR degeneration. | Cf02628333_m1 |
| *STK38L****** (*NRD2*) | Serine/threonine kinase 38 like | Regulates cell cycle, proliferation, and apoptosis. | Cf02709228_m1 (spans exons 4-5)  Cf02634613_m1 (spans exon 6-7) |

*****: subset of genes tested in RPE.

******: gene function information mainly based on GeneCards ([www.genecards.org](Supplemental%20Table%20S2%20genes-finale.docx)).
